# Contribution of Ca^2+^-Dependent Cl^−^ Channels to Norepinephrine-Induced Contraction of Femoral Artery Is Replaced by Increasing EDCF Contribution during Ageing

**DOI:** 10.1155/2014/289361

**Published:** 2014-02-23

**Authors:** Silvia Liskova, Miriam Petrova, Petr Karen, Michal Behuliak, Josef Zicha

**Affiliations:** ^1^Institute of Physiology, Academy of Sciences of the Czech Republic, 14220 Prague 4, Czech Republic; ^2^Institute of Pharmacology, Faculty of Medicine, Comenius University, Bratislava, Slovakia

## Abstract

The activation of Ca^2+^-dependent Cl^−^ channels during norepinephrine-induced contraction of vascular smooth muscle was suggested to depolarize cell membrane and to increase Ca^2+^ entry. Hypertension and ageing are associated with altered Ca^2+^ handling including possible activation of Ca^2+^-dependent Cl^−^ channels. Our study was aimed to determine Ca^2+^-dependent Cl^−^ channels contribution to norepinephrine-induced contraction during hypertension and ageing. Norepinephrine-induced concentration-response curves of femoral arteries from 6- and 12-month-old spontaneously hypertensive rats (SHR) and Wistar-Kyoto (WKY) rats were recorded using wire myograph. Pretreatment with Ca^2+^-dependent Cl- channel inhibitor indanyloxyacetic acid 94 [R(+)-IAA-94](IAA) attenuated norepinephrine-induced contraction in all groups, but relatively more in WKY than SHR arteries. The attenuation of norepinephrine-induced contraction after Ca^2+^-dependent Cl^−^ channels blockade was partially reduced in 12-month-old WKY rats, but substantially diminished in 12-month-old SHR. IAA effect was enhanced after NO synthase inhibition but decreased by ageing. In 20-month-old WKY rats norepinephrine-induced contraction was not affected by IAA but was almost abolished after cyclooxygenase inhibition by indomethacin or niflumic acid. In conclusion, contribution of Ca^2+^-dependent Cl^−^ channels to norepinephrine-induced contraction diminished with age, hypertension development, and/or NO synthesis inhibition. Ca^2+^-dependent Cl^−^ channels are important for maintenance of normal vascular tone while their inactivation/closing might be a pathological mechanism.

## 1. Introduction

The stimulation of vascular smooth muscle cells with norepinephrine leads to the release of Ca^2+^ from intracellular Ca^2+^ stores through IP_3_ pathway (phasic contraction) and thereafter to Ca^2+^ influx through the opening of L-type voltage-dependent Ca^2+^ channels (L-VDCC) (tonic contraction). The link between the opening of Ca^2+^ stores and L-VDCC could be mediated either through the elevation of intracellular Ca^2+^  [Ca^2+^]_*i*_ levels or might involve other mechanisms such as opening of Cl^−^ channels or closure of K^+^ channels [1–3]. Brayden and Nelson [4] showed that pressure-induced contraction of vascular smooth muscle is mediated by Ca^2+^ influx through L-VDCC and is modulated by a negative feedback pathway involving activation of large-conductance Ca^2+^-dependent K^+^ channels. We demonstrated that these K^+^ channels play an important role in modulating vascular contraction in spontaneous hypertension [5]. Calculated equilibrium potential for Cl^−^ in vascular cells is −26 mV [6], which is high enough to activate L-VDCC [7]. Furthermore, Ca^2+^-dependent Cl^−^ channels play a role in Ca^2+^ uptake to sarcoplasmic reticulum in smooth muscle because the administration of Cl^−^ channel blockers NPPB (5-nitro-2-(3-phenylpropylamino)benzoic acid) and IAA (indanyloxyacetic acid 94 (R(+)-IAA-94) almost completely blocked the Ca^2+^ uptake [8]. It is evident that Cl^−^ conductance is activated by the enhancement of either Ca^2+^ release from Ca^2+^ stores or Ca^2+^ influx [9]. The Ca^2+^-dependent Cl^−^ channels exist in two states, open and closed, with a relatively long mean open time. Some of the agents that inhibit Ca^2+^-dependent Cl^−^ channels interact directly with the open channel, which suggests that the most likely role of Ca^2+^-dependent Cl^−^ channels in smooth muscle is to produce membrane depolarization and contraction to neurotransmitters [10]. Therefore norepinephrine-induced contraction, which involves the increase of both Ca^2+^ release from Ca^2+^ stores and Ca^2+^ influx should be altered by the blockade of Ca^2+^-dependent Cl^−^ channels. It has been shown that Ca^2+^-dependent Cl^−^ channels play a major role in the response to norepinephrine in the portal vein [9] and mesenteric vein [11] or in the response of coronary and mesenteric arteries to endothelin [12]. Chloride channel blockers markedly attenuate both rapid and sustained responses of angiotensin II-induced contraction of renal vascular smooth muscle cells and thus contribute to, rather than being the consequence of, the initial rapid contractile response [13]. Pacaud et al. [14] suggested that the activation of Cl^−^ channels by Ca^2+^ release from intracellular stores might determine the maximal contraction by influencing the magnitude of membrane depolarization during agonist-induced contraction.

The activation of Ca^2+^-dependent Cl^−^ channels in aortic smooth muscle cells occurs after caffeine-induced Ca^2+^ release without Ca^2+^ influx, but the repetitive activation of Cl^−^ channels depends on Ca^2+^ influx through L-VDCC. Moreover, the progressive disappearance of caffeine-induced Ca^2+^ release and Ca^2+^-dependent Cl^−^ currents in smooth muscle cells treated with NO donors could result from NO-induced inhibition of ryanodine- and caffeine-sensitive Ca^2+^ channels and/or L-VDCC [15]. This mechanism could be more important for L-NAME-induced contraction of rat coronary arteries, because the changes of Cl^−^ conductance are involved in the L-NAME-induced contraction and the blockade of Cl^−^ channels abolished the L-NAME-induced contractions [16]. On the other hand, there are still confusing results about the involvement of endothelium in the effect of Cl^−^ channel activation. Using small mesenteric arteries Matchkov et al. [17] and Boedtkjer et al. [18] showed that there is a Cl^−^ current which is dependent on Ca^2+^ and cGMP and that the NO is necessary for the activation of this current, which leads to vasomotion and can participate in smooth muscle cell synchronization. This Ca^2+^-dependent and cGMP-dependent Cl^−^ current is sensitive to Zn^2+^ ions but less sensitive to conventional Cl^−^ channel blockers.

There are numerous reports on altered vascular function in hypertension and/or ageing in rats (for review see [19]). These alterations involve impaired endothelium-dependent vascular relaxation due to the attenuated vasodilation induced by nitric oxide [20, 21], *β*-adrenoceptor stimulation [22, 23], changes in Ca^2+^ entry and cell Ca^2+^ handling [24, 25], and so forth. Prominent role of Ca^2+^ influx through L-VDCC in the control of vascular tone and blood pressure can be demonstrated both under the *in vivo* and *in vitro* conditions as we have previously reported in conscious SHR and their isolated femoral arteries [26–28].

Our aim was to quantify the changes in Ca^2+^-dependent Cl^−^ channel-sensitive component of norepinephrine-induced contraction during the ageing and hypertension. Our hypothesis was that norepinephrine-stimulation of vascular smooth muscle opens/activates Ca^2+^-dependent Cl^−^ channels in SHR vessels to a larger extent than in those of WKY. The NO synthase inhibition was used to augment the magnitude of norepinephrine-induced contraction as well as to modify the contribution of the above ion channels to this contraction. IAA was used to inhibit Ca^2+^-dependent Cl^−^ channels in femoral arteries isolated from four groups of rats—adult WKY rats, aged WKY rats, adult SHR, and aged SHR. The second aim of our study was to compare the contribution of Ca^2+^-dependent Cl^−^ channels and endothelium-derived constricting factor (EDCF) to norepinephrine-induced contraction (including their age-dependent changes). This was possible to examine only in femoral arteries because EDCF is produced in femoral but not in small mesenteric arteries [29]. We investigated femoral arteries of 20-month-old WKY rats after the blockade of Ca^2+^-dependent Cl^−^ channels by IAA and after the inhibition of cyclooxygenase by indomethacin, which is the main enzyme producing endothelium-derived constricting factor (EDCF).

## 2. Material and Methods

### 2.1. General Procedure

Wistar-Kyoto (WKY) rats and spontaneously hypertensive rats (SHR) were sacrificed at the age of 6 months (adult WKY rats and adult SHR) and 12 months (aged WKY rats and aged SHR rats). The second series of experiments was carried out in 20-month-old WKY rats. Animals were housed under standard laboratory conditions (temperature 23 ± 1°C, 12 h light-dark cycle, pelleted ST-1 diet, and tap water *ad libitum*). All procedures and experimental protocols, which were approved by the *Ethical Committee of the Institute of Physiology, Academy of Sciences of Czech Republic*, conform to the *European Convention on Animal Protection* and *Guidelines on Research Animal Use*.

Animals were anesthetized with ether and blood pressure was measured directly by the puncture of carotid artery. The animals were killed by overdose of CO_2_ and after decapitation femoral arteries with intact endothelium were cut into 2 mm long segments and placed in a Mulvany-Halpern isometric myograph (M 510A, DMT, Denmark). The myograph chamber was filled with modified Krebs-Henseleit solution (119 mM NaCl, 4.7 mM KCl, 1.17 mM MgSO_4_, 25 mM NaHCO_3_, 1.18 mM KH_2_PO_4_, 2.5 mM CaCl_2_, 2 g/L glucose, 37°C) and bubbled with 95% O_2_ and 5% CO_2_. The inner arterial diameter was set to be 90% of the diameter predicted for the pressure of 100 mm Hg in the wire myograph. During 30 min of stabilization the vessels were left to achieve their basal tone. To provide the maximal depolarization-induced contraction vessels were incubated with a depolarizing solution (modified Krebs-Henseleit solution with 124 mM K^+^ but without Na^+^).

### 2.2. Experimental Protocol: The Role of Ca^2+^-Dependent Cl^−^ Channel in Norepinephrine-Induced Contraction

The femoral arteries isolated from adult and aged WKY rats and SHR were studied in the first part of our experiments. Subsequent arterial contractions were induced by cumulative doses of norepinephrine (3.10^−8^ to 10^−4^ mol/L) and then acetylcholine-induced relaxations (3.10^−8^ mol/L and 10^−6^ mol/L or 3.10^−6^ mol/L) of norepinephrine-precontracted vessels were measured. One segment with preserved endothelium was studied in each rat. After repeated washing and stabilization of basal tone norepinephrine-induced concentration-response curves were again determined in the same artery segment in the presence of NO synthase inhibitor N^*ω*^-nitro-L-arginine (L-NNA, 10^−4^ mol/L, preincubation time 15 min) or in the presence of Ca^2+^-dependent Cl^−^ channel blocker indanyloxyacetic acid 94 (R(+)-IAA-94, IAA, 10^−5^ mol/L, preincubation time 10 min). Finally, these concentration-response curves were measured in the presence of both inhibitors L-NNA and IAA.

### 2.3. Experimental Protocol: The Changes of Ca^2+^-Dependent Cl^−^ Channels and EDCF during Ageing

In the second part of our study we used femoral arteries isolated from 20-month-old WKY rats. Arterial contraction induced by cumulative doses of norepinephrine (10^−8^ to 10^−4^ mol/L) followed by arterial relaxation elicited by acetylcholine application (10^−8^ mol/L and 10^−6^ mol/L) were first measured in the absence of inhibitors. Thereafter norepinephrine-induced concentration-response curves were determined in the presence of Ca^2+^-dependent Cl^−^ channel blocker indanyloxyacetic acid 94 (R(+)-IAA-94, IAA, 10^−5^ mol/L, preincubation time 10 min) and after 30 min washing period again in the presence of cyclooxygenase inhibitor indomethacin (IME, 10^−5^ mol/L, preincubation time 10 min). In another group of vessels norepinephrine-induced concentration-response curves were determined in the presence of niflumic acid (NIFLU, 10^−5^ mol/L, preincubation time 10 min) which was used as cyclooxygenase inhibitor and Ca^2+^-dependent Cl^−^ channel blocker.

### 2.4. Drugs and Statistical Analysis

All chemicals were obtained from Sigma (Heidelberg, Germany). R(+)-IAA-94 and niflumic acid were dissolved in ethanol maintaining the final concentration of ethanol in the chamber under 1%. Indomethacin was dissolved in 170 mM Na_2_CO_3_. Logistic model was used for fitting concentration-response curves through the measure values [30]. Pharmacodynamic characteristics—EC50 (half-maximal effective concentration, log mol/L), *E*_max⁡_ (maximal contraction, mN/mm), and slope—were calculated for each norepinephrine-induced concentration-response curve. Data are presented as mean ± S.E.M. Statistical analysis was performed with one-way ANOVA and *post hoc* least significant difference test.

## 3. Results

### 3.1. Basal Characteristics

Blood pressure was higher in SHR than WKY rats without any significant age-dependent increase. The inner diameter of studied femoral arteries was always significantly smaller in SHR than in WKY rats, but it increased with age in both rat strains (Table 1).

### 3.2. Vascular Responses of Femoral Arteries in Adult and Aged WKY or SHR

**Figure 1:**
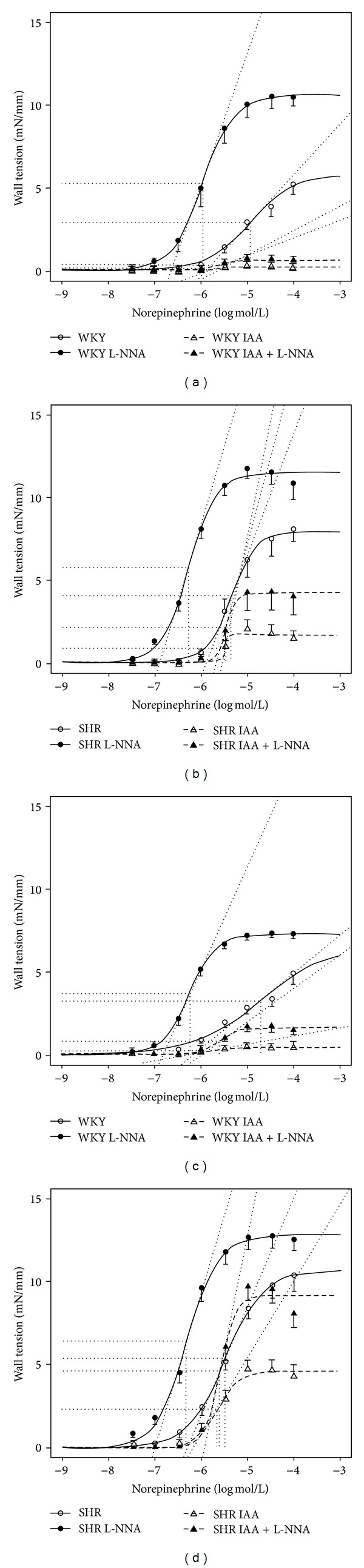
Norepinephrine concentration-response curves obtained in femoral arteries of adult WKY rats (6 months old, (a)), adult SHR (6 months old, (b)), aged WKY rats (12 months old, (c)), and aged SHR (12 months old, (d)) recorded under the control conditions and after the inhibition of NO synthase (L-NNA) or Ca^2+^-dependent Cl^−^ channels blockade (R(+)-IAA-94, IAA) or both (IAA + L-NNA). Data are presented as mean ± S.E.M. (for number of vessels see Table 1). Depicted curves were calculated from average values obtained at studied norepinephrine concentrations.

The administration of cumulative norepinephrine concentrations to isolated femoral arteries yielded concentration-response curves which were similar in vessels from adult WKY and aged WKY. As far as concentration-response curves of SHR arteries are concerned, the maximal contraction (*E*_max⁡_) was enhanced in vessels from SHR compared to WKY vessels. Ageing increased the maximal contraction in vessels from SHR only (Figure 1; Table 2). Acetylcholine at the concentration 3.10^−8^ mol/L relaxed the arteries of adult and aged WKY as well as those of adult SHR by about 50%. The vessels from aged SHR relaxed by 37% only (Table 3). At the higher concentrations of acetylcholine (10^−6^ mol/L in WKY and 3.10^−6^ mol/L in SHR) the maximal acetylcholine-induced relaxation of isolated femoral arteries was only slightly attenuated in SHR arteries (Table 3). The wall tension decrease after acetylcholine was greater in SHR vessels, which corresponds to the enhanced arterial contraction in SHR.

### 3.3. Effect of Ca^2+^-Dependent Cl^−^ Channels Inhibition on Norepinephrine-Induced Concentration-Response Curves

The inhibition of Ca^2+^-dependent Cl^−^ channels with IAA almost abolished the norepinephrine-induced contraction of vessels from adult and aged WKY rats and largely reduced the contraction of vessels from adult SHR. The relative inhibitory effect of IAA was surprisingly smallest in femoral arteries isolated from aged SHR (Figure 1; Table 2). The relative contribution of Ca^2+^-dependent Cl^−^ channel-sensitive component to norepinephrine-induced contraction was 97% in adult WKY, 90% in aged WKY, 80% in adult SHR, but only 55% in aged SHR. The sensitivity to norepinephrine (EC50) was enhanced and *E*_max⁡_ was impaired by IAA pretreatment of WKY vessels compared to the control conditions. In contrast, there were no significant changes in norepinephrine sensitivity of concentration-response curves in SHR vessels (Table 2).

### 3.4. Effect of Ca^2+^-Dependent Cl^−^ Channels Inhibition on Norepinephrine-Induced Concentration-Response Curves during NO Synthase Inhibition

The inhibition of NO synthase with L-NNA shifted the concentration-response curves to the left in all groups (Figure 1). *E*_max⁡_ was substantially increased as compared to the control conditions (Table 2). In the absence of NO synthesis the inhibition of Ca^2+^-dependent Cl^−^ channels shifted norepinephrine-induced concentration-response curves to the right (Figure 1) and reduced *E*_max⁡_ more in WKY than in SHR vessels (Table 2). The relative contribution of Ca^2+^-dependent Cl^−^ channel-sensitive component represented 93% in adult WKY and 78% in aged WKY,but only 63% in adult SHR and 28% of norepinephrine-induced contraction in aged SHR. Thus, the effect of IAA was again smallest in aged SHR.

### 3.5. The Changes between Ca^2+^-Dependent Cl^−^ Channels and EDCF during Ageing

Norepinephrine-induced contraction of vessels isolated from 20-month-old WKY rats were slightly more sensitive to norepinephrine and reached slightly higher maximal contraction values as compared to adult and aged WKY rats. The acetylcholine-induced relaxation was impaired at low concentration of acetylcholine, but it was not changed at higher acetylcholine concentration and reached 79 ± 2% of norepinephrine precontraction (Table 3).

**Figure 2:**
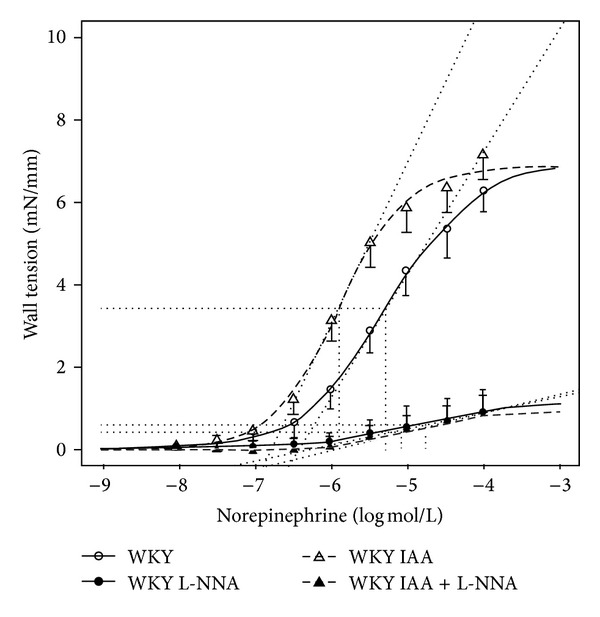
Norepinephrine concentration-response curves obtained in femoral arteries of 20-month-old WKY rats (WKY) recorded under the control conditions and after Ca^2+^-dependent Cl^−^ channels blockade (R(+)-IAA-94, IAA) or after the inhibition of cyclooxygenase (indomethacin, IME) or in the presence of niflumic acid (NIFLU). Data are presented as mean ± S.E.M. (for number of vessels see Table 4). Depicted curves were calculated from average values obtained at studied norepinephrine concentrations.

Surprisingly, the application of Ca^2+^-dependent Cl^−^ channel blocker IAA did not impair the norepinephrine-induced contraction as it was shown in our previous experiments with 6- and 12-month-old rats. As in previous experiments with 6- and 12-month-old rats IAA increased EC50, but the *E*_max⁡_ was not altered significantly. The subsequent inhibition of cyclooxygenase with indomethacin almost fully abolished the norepinephrine-induced contraction. In parallel experiments the application of niflumic acid, which is considered to be Ca^2+^-dependent Cl^−^ channel blocker and cyclooxygenase inhibitor, also abolished the norepinephrine-induced contraction to a similar extent as indomethacin. Our experiments have clearly shown that niflumic acid is a potent cyclooxygenase inhibitor, which cannot be used as a simple Ca^2+^-dependent Cl^−^ channel blocker (Table 4; Figure 2).

## 4. Discussion

We have investigated the effect of ageing and hypertension on the participation of Ca^2+^-dependent Cl^−^ channels in norepinephrine-induced contraction of femoral arteries. When evaluating Ca^2+^-dependent Cl^−^ channel-sensitive component of norepinephrine-induced contraction we have demonstrated that the inhibition of Ca^2+^-dependent Cl^−^ channels largely impaired the norepinephrine-induced contraction of femoral arteries in all studied groups of rats. Thus, our data are in good agreement with the earlier findings on norepinephrine-induced increase of Cl^−^ conductance in portal vein smooth muscle cells [9]. However, the relative effects were greatest in the arteries of adult WKY rats but smallest in vessels of aged SHR. Our results showed that the contribution of Ca^2+^-dependent Cl^−^ channels to norepinephrine-induced contraction is reduced during ageing, and hypertension further enhanced this reduction. The mechanism(s) responsible for the residual part of norepinephrine-induced arterial contraction seen mainly in SHR vessels remains to be determined. Resting membrane potential of vascular smooth muscle cells of SHR is increasing during the hypertension development and stays significantly higher compared to normotensive WKY rats [31]. Contrary to our expectations, norepinephrine stimulation of vascular smooth muscle did not open/activate Ca^2+^-dependent Cl^−^ channels to a larger extent in vascular smooth muscle of SHR.

IAA is a potent Ca^2+^-dependent Cl^−^ channel inhibitor, but it has also a relatively high affinity to L-VDCC [32]. It is unlikely that IAA affected the L-VDCC significantly in our experiments because a greater IAA effect was disclosed in vessels from adult WKY compared to those from SHR, although L-VDCC plays a more important role in SHR than in WKY [24]. Ca^2+^-dependent Cl^−^ channels blockers also activate large-conductance Ca^2+^-activated K^+^ channels [33]. The concentration used in our experiments was lower as assumed for the activation of Ca^2+^-activated K^+^ channels (10^−5^ mol/L versus 5 × 10^−4^ mol/L, [34]). To our knowledge there is no evidence about decreasing influence of Ca^2+^-activated K^+^ channels during ageing and further experiments are needed to show if the effect of IAA seen in our study was partially through Ca^2+^-activated K^+^ channels.Niflumic acid is also a potent cyclooxygenase inhibitor. A comparison of the effect of indomethacin and niflumic acid clearly shows that both compounds are acting through the inhibition of cyclooxygenase pathway because it was impossible to produce additive changes in contraction by the combination of these two compounds.

We have recently demonstrated [35] that a considerable part of norepinephrine-induced contraction of rat femoral artery was mediated by endothelium-derived constricting factor (EDCF). This type of arterial contraction can be largely prevented by the pretreatment of vessels with cyclooxygenase inhibitor indomethacin. Indomethacin administration was also capable of inducing a substantial reduction of the already developed arterial contraction. It is important to note that EDCF contribution to norepinephrine-induced arterial contraction was increasing with age and hypertension development [35]. The same was reported for the role of EDCF in the age-dependent impairment of acetylcholine-induced endothelium-dependent vascular relaxation in developing SHR [36, 37]. The mechanisms underlying the age-dependent increase of EDCF formation and/or action remain unclear. It can be speculated that the earlier described age-dependent elevation of intracellular Na^+^ concentration could augment cyclooxygenase 2 expression as it was demonstrated in cells with increased Na^+^_*i*_/K^+^_*i*_ ratio [38]. Nevertheless, it should be mentioned that EDCF participation in vascular contraction elicited by norepinephrine or high doses of acetylcholine is characteristic for conduit arteries because it is absent in resistance arteries such as small mesenteric vessels [29]. Thus the role of EDCF in blood pressure control remains questionable.

Ca^2+^-dependent Cl^−^ channels are involved in a major part of norepinephrine-induced contraction of adult and aged WKY arteries, but the contribution of these channels to norepinephrine-induced contraction is decreasing with age and/or hypertension development as was shown using the arteries of 20-month-old WKY rats in which the blockade of Ca^2+^-dependent Cl^−^ channels was without effect on norepinephrine-induced contraction (Figure 2). This is in contrast with the increasing role of cyclooxygenase-sensitive component of norepinephrine-induced contraction during ageing and hypertension [35]. Thus the participation of Cl^−^ channels in norepinephrine-induced arterial contraction was drastically reduced in aged SHR compared to adult animals, but it was not found in arteries of 20-month-old WKY rats. It seems that the role of Ca^2+^-dependent Cl^−^ channels is replaced by the increasing influence of EDCF (cyclooxygenase-sensitive component of norepinephrine-induced contraction). It remains to be determined which other cyclooxygenase-sensitive mechanism is responsible for the progressive age-dependent augmentation of norepinephrine-induced contraction of large arteries in ageing and hypertension.

In contrast to the lack of effects of Ca^2+^-dependent Cl^−^ channel inhibition, the inhibition of cyclooxygenase with indomethacin led to the attenuation of norepinephrine-induced contraction of femoral arteries of 20-month-old WKY rats. In parallel experiments with vessels of these old rats, the application of niflumic acid led to the attenuation of norepinephrine-induced contraction as well. Niflumic acid is considered to be a Ca^2+^-dependent Cl^−^ channel inhibitor as well as cyclooxygenase inhibitor. Our experiments clearly indicated that niflumic acid is a potent cyclooxygenase inhibitor and that the results obtained with niflumic acid in other studies should be considered carefully. Unfortunately, we were not able to investigate the effect of niflumic acid after the administration of indomethacin on norepinephrine-induced contraction because indomethacin alone almost abolished the contraction to norepinephrine.

The inhibition of NO synthesis pronounced the age- and hypertension-dependent reduction of Ca^2+^-dependent Cl^−^ channel-sensitive component of norepinephrine-induced contraction. It is possible that the endothelial factors (such as NO) might contribute to the reduction of IAA-sensitive component of norepinephrine-induced contraction. Further studies with more specific compounds are needed to separate the Cl^−^ current dependent on NO as shown by Matchkov et al. [17] and Boedtkjer et al. [18] from the Ca^2+^-dependent Cl^−^ current in vascular smooth muscle cells.

It is important to note that the inhibition of Ca^2+^-dependent Cl^−^ channels was suggested as a possible therapeutic target in hypertension [39]. In the present study we provide the first evidence that the activation/opening of Ca^2+^-dependent Cl^−^ channels is important for the maintenance of normal physiological state during norepinephrine-induced contraction, at least in femoral arteries. Further attention should be paid to the interactions of Cl^−^ channels with other ion transporter systems. Recent data indicate that Cl^−^ channels in vascular smooth muscle cells are under the control of inwardly directed Na^+^, K^+^, and 2Cl^−^ cotransport (NKCC1). Numerous research groups demonstrated that the inhibitors of this carrier such as bumetanide and furosemide sharply diminished contraction of blood vessels elicited by norepinephrine or other vasoconstrictors (for review see [40]). Increased *nkcc1* mRNA expression and NKCC1 protein content in the aorta of SHR was accompanied by hypomethylation of the *nkcc1* gene promoter [41]. It is important to note that the methylation of *nkcc1* promoter in normotensive WKY rats was increased with age, whereas in SHR it remained hypomethylated after hypertension development. Both increased *nkcc1* expression and inhibitory action of bumetanide on mesenteric artery contractions were increased with age in SHR but not in WKY rats [42]. It would therefore be desirable to study the effects of Ca^2+^-dependent Cl^−^ channel blockers on norepinephrine-induced vascular contraction in the presence of NKCC1 inhbitors.

In conclusion, our study demonstrated a considerable contribution of Ca^2+^-dependent Cl^−^ channels to norepinephrine-induced arterial contraction, which diminishes with age, hypertension development, and/or inhibition of NO synthesis. Further studies are needed to provide the evidence for our hypothesis that Ca^2+^-dependent Cl^−^ channels are important for the maintenance of normal physiological state in the vascular system. The loss or inactivation of these channels during ageing and/or hypertension development could be seen as a pathological mechanism.

## 5. Limitations of the Study

The contribution of Ca^2+^-dependent Cl^−^ channels in norepinephrine-induced arterial contraction should be investigated in young normotensive and genetically hypertensive rats to reveal their potential role in the development of hypertension. The contribution of these ion channels to arterial contraction elicited by other vasoconstrictors (such as angiotensin II or endothelin-1) should also be studied. It would be desirable to evaluate the above mechanism in other forms of experimental hypertension.

## Figures and Tables

**Table 1:** Blood pressure and the diameter of isolated femoral arteries in adult and aged Wistar-Kyoto rats (WKY) and spontaneously hypertensive rats (SHR).

Parameters	Adult WKY (6 months) (*n* = 6)	Aged WKY (12 months) (*n* = 7)	Adult SHR (6 months) (*n* = 6)	Aged SHR (12 months) (*n* = 9)
SBP (mm Hg)	131 ± 3	134 ± 4	221 ± 5^a^	215 ± 6^c^
MAP (mm Hg)	109 ± 3	112 ± 3	182 ± 5^a^	187 ± 4^c^
DBP (mmHg)	91 ± 3	94 ± 3	152 ± 4^a^	163 ± 4^c^
Diameter (*μ*m)	995 ± 21	1052 ± 20^a^	843 ± 27^a^	943 ± 17^b,c^

Data are presented as mean ± S.E.M. SBP: systolic blood pressure; MAP: mean arterial pressure; DBP: diastolic blood pressure. Significant differences: ^a^ *P* < 0.05 versus adult WKY; ^b^ *P* < 0.05 versus adult SHR; ^c^ *P* < 0.05 versus aged WKY.

**Table 2:** Pharmacodynamic parameters of norepinephrine concentration-response curves determined in individual femoral arteries isolated from adult and aged WKY and SHR which were studied under the control conditions (CONTROL), in the presence of N^*ω*^-nitro-L-arginine (L-NNA, NO synthase inhibitor) and in the presence of R(+)-IAA-94 (IAA, Ca^2+^-dependent Cl^−^ channel blocker) as well as in the presence of IAA and L-NNA (IAA + L-NNA).

	Parameters	Adult WKY (6 months)	Aged WKY (12 months)	Adult SHR (6 months)	Aged SHR (12 months)
CONTROL (6/7/6/9)	EC50 (log mol/L)	−4.93 ± 0.09	−5.11 ± 0.13	−5.12 ± 0.15	−5.43 ± 0.08
	*E*_max⁡_ (mN/mm)	5.26 ± 0.84	5.30 ± 0.64	8.25 ± 0.32^a^	10.41 ± 0.70^b,c^
	Slope	3.69 ± 0.55	3.11 ± 0.77	11.27 ± 0.52^a^	8.80 ± 1.10^c^

L-NNA (6/7/6/7)	EC50 (log mol/L)	−5.84 ± 0.13^d,e^	−6.14 ± 0.07^d,e^	−6.17 ± 0.04^d,e^	−6.28 ± 0.07^d,e^
	*E*_max⁡_ (mN/mm)	10.48 ± 0.70^d,e^	7.29 ± 0.30^a,d,e^	11.31 ± 0.54^d,e^	12.63 ± 0.78^c,d,e^
	Slope	10.03 ± 1.60^d^	9.77 ± 1.84	12.24 ± 0.46	15.71 ± 3.12

IAA (6/7/6/9)	EC50 (log mol/L)	−5.53 ± 0.13^d^	−5.79 ± 0.13^d,e^	−5.35 ± 0.05	−5.48 ± 0.07
	*E*_max⁡_ (mN/mm)	0.15 ± 0.05^d^	0.53 ± 0.08^d^	1.68 ± 0.20^a,d,e^	4.66 ± 0.60^b,c,d,e^
	Slope	2.63 ± 0.78	1.68 ± 0.12	9.50 ± 1.96	21.12 ± 7.04

IAA + L-NNA (6/7/6/7)	EC50 (log mol/L)	−5.42 ± 0.12^d^	−5.47 ± 0.08^d^	−5.28 ± 0.02	−5.46 ± 0.07^b^
	*E*_max⁡_ (mN/mm)	0.76 ± 0.03^d^	1.64 ± 0.38^d^	4.21 ± 0.52^a,d^	9.17 ± 0.90^b,c^
	Slope	2.00 ± 0.77	6.29 ± 3.20	18.40 ± 4.49^a^	14.03 ± 0.76

Data are presented as mean ± S.E.M., the number of vessels studied under different experimental conditions is indicated in parentheses (adult WKY/aged WKY/adult SHR/aged SHR). Significant differences: ^a^ *P* < 0.05 versus adult WKY; ^b^ *P* < 0.05 versus adult SHR; ^c^ *P* < 0.05 versus aged WKY; ^d^ *P* < 0.05 versus Control; ^e^ *P* < 0.05 versus IAA + L-NNA.

**Table 3:** Maximal norepinephrine-induced contraction and acetylcholine-induced relaxation of femoral arteries isolated from adult and aged WKY and SHR.

Parameters	Adult WKY (6 months) (*n* = 6)	Aged WKY (12 months) (*n* = 7)	Adult SHR (6 months) (*n* = 6)	Aged SHR (12 months) (*n* = 9)	WKY (20 months) (*n* = 16)
Norepinephrine-induced (10^−4^ mol/L) maximal contraction (mN/mm)	5.26 ± 0.84	5.30 ± 0.64	8.25 ± 0.32^a^	10.41 ± 0.70^a,b^	6.79 ± 0.20
Acetylcholine-induced (3.10^−8^ mol/L) relaxation (%)	53 ± 3	47 ± 6	55 ± 4	37 ± 4^a,b^	18 ± 4^c^
Acetylcholine-induced (3.10^−8^ mol/L) wall tension decrease (mN/mm)	−2.70 ± 0.36	−2.02 ± 0.41	−4.37 ± 0.70^(a)^	−4.18 ± 0.55^a^	−0.98 ± 0.22^c^
Acetylcholine-induced relaxation (%)*	85 ± 4	82 ± 5	67 ± 4^a^	70 ± 2	79 ± 2
Acetylcholine-induced wall tension decrease (mN/mm)*	−4.37 ± 0.65	−3.49 ± 0.44	−5.30 ± 0.38	−8.04 ± 0.84^a,b^	−4.70 ± 0.23

Data are presented as mean ± S.E.M.; ^a^ *P* < 0.05 versus aged-matched WKY; ^b^ *P* < 0.05 versus adult SHR; ^c^ *P* < 0.05 versus WKY; ^(a)^represents borderline significance *P* < 0.08. *Maximal acetylcholine-induced relaxations were achieved in femoral arteries of WKY at the concentration 10^−6^ mol/L and in femoral arteries of SHR at the concentration 3.10^−6^ mol/L.

**Table 4:** Pharmacodynamic parameters of norepinephrine concentration-response curves determined in individual femoral arteries isolated from 20-month-old WKY rats in the presence of R(+)-IAA-94 (IAA, Ca^2+^-dependent Cl^−^ channel blocker) and indomethacin (IME, cyclooxygenase inhibitor) as well as in the presence of niflumic acid (NIFLU, cyclooxygenase inhibitor, and Cl^−^ channel blocker).

	Parameters	Control (*n* = 16)	IAA (*n* = 8)	IME (*n* = 8)	NIFLU (*n* = 8)
WKY	EC50 (log mol/L)	−5.22 ± 0.12	−5.89 ± 0.10^a^	−4.67 ± 0.18^b^	−4.93 ± 0.18^b^
	*E*_max⁡_ (mN/mm)	6.79 ± 0.20	6.86 ± 0.26	1.40 ± 0.10^a,b^	1.18 ± 0.06^a,b^
	Slope	3.90 ± 0.37	4.49 ± 0.35	0.43 ± 0.10^a,b^	0.63 ± 0.09^a,b^

Data are presented as mean ± S.E.M.; ^a^ *P* < 0.05 versus control; ^b^ *P* < 0.05 versus IAA.
