# Erratum to “Contribution of Ca^2+^-Dependent Cl^−^ Channels to Norepinephrine-Induced Contraction of Femoral Artery Is Replaced by Increasing EDCF Contribution during Ageing”

**DOI:** 10.1155/2018/3081532

**Published:** 2018-02-20

**Authors:** Silvia Liskova, Miriam Petrova, Petr Karen, Michal Behuliak, Josef Zicha

**Affiliations:** ^1^Institute of Physiology, Academy of Sciences of the Czech Republic, 14220 Prague 4, Czech Republic; ^2^Institute of Pharmacology, Faculty of Medicine, Comenius University, Bratislava, Slovakia

 In the article titled “Contribution of Ca^2+^-Dependent Cl^−^ Channels to Norepinephrine-Induced Contraction of Femoral Artery Is Replaced by Increasing EDCF Contribution during Ageing” [1], there was an error in the key of Figure 2, which is corrected as follows.

## Figures and Tables

**Figure 2:**
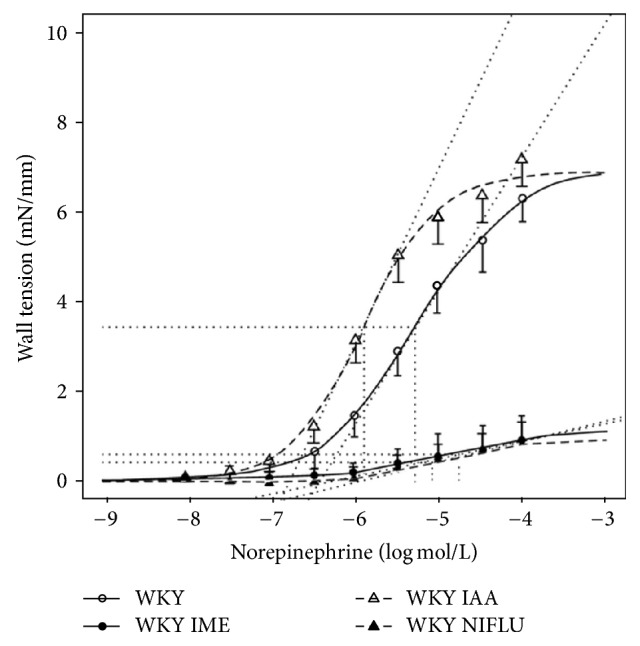
Norepinephrine concentration-response curves obtained in femoral arteries of 20-month-old WKY rats (WKY) recorded under the control conditions and after Ca^2+^-dependent Cl^−^ channels blockade (R(+)-IAA-94, IAA) or after the inhibition of cyclooxygenase (indomethacin, IME) or in the presence of niflumic acid (NIFLU). Data are presented as mean ± SEM (for number of vessels see Table 4). Depicted curves were calculated from average values obtained at studied norepinephrine concentrations.

## References

[1] Liskova S., Petrova M., Karen P., Behuliak M., Zicha J. (2014). Contribution of Ca^2+^-dependent Cl^−^ channels to norepinephrine-induced contraction of femoral artery is replaced by increasing EDCF contribution during ageing. *BioMed Research International*.

